# Neuromuscular Activity Induces Paracrine Signaling and Triggers Axonal Regrowth after Injury in Microfluidic Lab-On-Chip Devices

**DOI:** 10.3390/cells9020302

**Published:** 2020-01-27

**Authors:** Julia Sala-Jarque, Francina Mesquida-Veny, Maider Badiola-Mateos, Josep Samitier, Arnau Hervera, José Antonio del Río

**Affiliations:** 1 Institute for Bioengineering of Catalonia (IBEC), The Barcelona Institute of Science and Technology, 08028 Barcelona, Spain; jsala@ibecbarcelona.eu (J.S.-J.); fmesquida@ibecbarcelona.eu (F.M.-V.); mbadiola@ibecbarcelona.eu (M.B.-M.); jsamitier@ibecbarcelona.eu (J.S.); 2 Centro de Investigación Biomédica en Red sobre Enfermedades Neurodegenerativas (CIBERNED), 28031 Madrid, Spain; 3 Department of Cell Biology, Physiology and Immunology, Faculty of Biology, Universitat de Barcelona, 08028 Barcelona, Spain; 4 Institute of Neuroscience, University of Barcelona, 08028 Barcelona, Spain; 5 Centro de Investigación Biomédica en Red en Bioingeniería, Biomateriales y Nanomedicina (CIBERBBN), 28029 Madrid, Spain; 6 Department of Electronics and Biomedical Engineering, Universitat de Barcelona, 08028 Barcelona, Spain

**Keywords:** neuromuscular junction, microfluidics, axotomy, paracrine signaling

## Abstract

Peripheral nerve injuries, including motor neuron axonal injury, often lead to functional impairments. Current therapies are mostly limited to surgical intervention after lesion, yet these interventions have limited success in restoring functionality. Current activity-based therapies after axonal injuries are based on trial-error approaches in which the details of the underlying cellular and molecular processes are largely unknown. Here we show the effects of the modulation of both neuronal and muscular activity with optogenetic approaches to assess the regenerative capacity of cultured motor neuron (MN) after lesion in a compartmentalized microfluidic-assisted axotomy device. With increased neuronal activity, we observed an increase in the ratio of regrowing axons after injury in our peripheral-injury model. Moreover, increasing muscular activity induces the liberation of leukemia inhibitory factor and glial cell line-derived neurotrophic factor in a paracrine fashion that in turn triggers axonal regrowth of lesioned MN in our 3D hydrogel cultures. The relevance of our findings as well as the novel approaches used in this study could be useful not only after axotomy events but also in diseases affecting MN survival.

## 1. Introduction

Peripheral nerve (PN) injuries, including motor neuron (MN) axonal injury, are a major problem confronting modern rehabilitation medicine. In fact, current therapies are mostly limited to surgical intervention after lesion. Unfortunately, the success rate of such interventions has not improved since the introduction of enhanced microsurgical techniques several decades ago. Moreover, current activity-based therapies after axonal injuries have only limited success, and most of them are trial–error or proof-of-concept approaches in which the details of the underlying cellular and molecular processes are largely unknown. Therefore, it is mandatory to develop new strategies focused on identifying novel targets that may foster axonal regeneration and functional recovery following PN axonal injuries. This can only be achieved with clear monitoring of the molecular and biological events that occur in lesioned neurons and target cells after lesion. This will in turn help identify new targets and cues to unravel the precise mechanisms underlying neuronal activity and axon growth dynamics after injury. 

Since the implementation of the compartmentalized neuronal cell culture [1] years ago [2,3], microfluidic technology, micropatterning, and microfabrication allowing spatial and fluidic segregation of neuronal soma from axons [4] have greatly improved. This technology has permitted real-time monitoring of physiological events, thus becoming a key reference technique for many researchers and laboratories (see also [5,6] for review). Additionally, some studies have also reported different ways to lesion axons on lab-on-chip devices (i.e., [7]). However, these studies mainly used the devices to support in vivo experimentation and did not take advantage of the full potential of these platforms (see [8] for a recent example). Concerning PN-modelling, several methods were developed to generate contractile myotubes on lab-on-chip devices (i.e., [9,10,11]). But more relevantly, numerous protocols focused on mimicking PN networks (allowing neuromuscular junction (NMJ) formation) using muscle-derived cells (i.e, [12]), spinal cord slices (i.e., [13]), stem cells (i.e., [14,15]), and induced pluripotent stem cells (i.e., [16]), and in some of these, muscle activity was also analyzed (i.e., [11,16,17,18]). However, none of these methods monitor the molecular events that occur in both muscle cells and MN after a PN axotomy. So, in this study, we took advantage of the microfluidic co-culture of ventral horns of spinal cord and muscle cells to determine factors secreted by muscle cells with positive function in MN axonal regrowth. Indeed, although the molecular mechanisms underlying functional PN axon regeneration remain poorly understood, growing evidence suggests that fine cellular, spatial, and temporal control of cellular and paracrine signaling during regenerative events is crucial. Growing evidence has led to the hypothesis that paracrine signaling from neighboring cells or target cells (i.e., Schwann cells or muscles, respectively) might play a role (positive or negative) during the regenerative events after PN lesion. These signals include developmentally-regulated molecules such as class III secreted semaphorins and their plexin receptors [19,20] as well as membrane-anchored molecules. Pioneering studies point to the crucial roles of the L2/HNK-1 membrane carbohydrate (preferentially expressed by motor axon-associated Schwann cells, and associated with cell adhesion) during PN regeneration [21,22,23]. Indeed, some studies have shown that different molecules might trigger specifically motor or sensorial axonal growth (i.e., muscle-derived Brain-derived neurotrophic factor (BDNF) [24] or Schwann cell-derived molecules [25]). Several studies demonstrate an increase of growth factor expression in muscle as a consequence of denervation [26,27,28,29,30,31,32]; indeed, conditioned media from muscle can act as a trophic factor for motor neurons [33]. However, little is known about the effect of induced neuromuscular activity after muscle denervation on lesioned MN axons. In this study, we show the effects of the modulation of both neuronal and muscular activity with optogenetic approaches to assess the regeneration ability of cultured MN after lesion in a compartmentalized microfluidic-assisted axotomy device. We first show that increasing neuronal activity enhances the ratio of regenerative axons after injury in our model. In a second set of experiments, we demonstrate that increasing muscular activity after MN axotomy by optical stimulation in our platform induces the secretion of particular paracrine factors. Of these, leukemia inhibitory factor (LIF) and glial cell line-derived neurotrophic factor (GDNF) trigger axonal regrowth of lesioned MN in 3D hydrogel cultures.

## 2. Materials and Methods

### 2.1. Animals

Embryonic day 12.5 (E12.5) pregnant CD1 female mice were obtained from Charles River Laboratories (France). All experiments were performed under the guidelines and protocols of the Ethical Committee for Animal Experimentation (CEEA) of the University of Barcelona, and the protocol for the use of animals in this study was reviewed and approved by the CEEA of the University of Barcelona (CEEA approval #276/16 and #141/15).

### 2.2. Compartmentalized Microfluidic Devices

**Figure 1:**
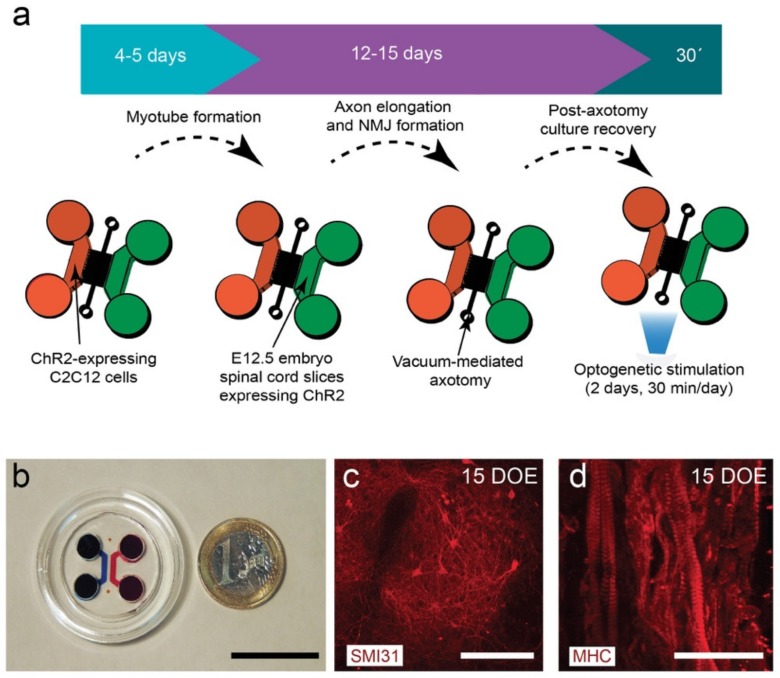
(**a**) General pipeline of the establishment of an optogenetically modular neuromuscular junction (NMJ) compartmentalized microfluidic platform (see Material and Methods for details). (**b**) Low magnification photomicrograph of the assembled microfluidic device. (**c**) SMI31-positive neurons in a spinal cord explant cultured for 15 days in the neuronal chamber during the second phase of the protocol (violet arrow in (a)). (**d**) Myosin heavy chain (MHC)-positive myotubes cultured for 15 days in the first phase of the protocol (blue arrow in (a)). Scale bars: (b) = 2.5 cm; (c) = 50 µm, and (d) = 75 µm.

Poly(dimethylsiloxane) (PDMS) microfluidic devices were designed in CAD software and chrome masks were prepared at IBEC Microfabspace. The lab-on-chip microfluidic device design was modified and adapted for our purposes from previously published devices [34,35]. The axotomy microfluidic device (Figure 1) consisted of 4 circular reservoirs of 7–8 mm of Ø interconnected by two cell/explant seeding chambers (150 (high) × 1,500 (wide) µm). Between these two chambers, a total of 100 microchannels of 10 (high) × 10 (wide) µm sections and 900 µm (length) were present. In addition, a perpendicular channel trespassing the microchannels (the axotomy channel) with two 1.25 mm Ø inlet and outlet ports was also included. The axotomy channel itself, located at a distance of 400 µm from each chamber, consisted of a 100 (high) × 100 (wide) µm section and a 12,000 µm (long) channel (Figure 1). The device master was made using standard photolithography and soft lithography techniques, as described before [36]. PDMS, mixed at 10:1 base/curing agent (*w/w*), was poured onto the master and cured at 80 °C for at least 4 h. Devices were then cut off the mold and trimmed to the appropriate size; afterwards, the different ports were formed with 1.25 and 7 mm Ø biopsy punches. Following this, the microfluidic devices were subjected to sterilization and permanently bonded to Nunc™ Glass Bottom 35 mm ∅ dishes (ThermoFisher Scientific, USA) with oxygen plasma treatment. Once ready, the mounted devices were sterilized once more by placing them under ultraviolet (UV) light inside a culture hood for 1 h. In all experiments and conditions, before cell/explant seeding, the culture surface of the device was pre-treated with 1:50 Matrigel™ dilution in Dulbecco’s modified Eagle’s medium (DMEM, ThermoFisher Scientific, Spain) at 4 °C overnight. The following day, devices were kept for 30 min at 37 °C with a 5% CO_2_ atmosphere.

### 2.3. C2C12 Myoblast Culture and Generation of a Stable ChR2-eYFP-Expressing C2C12 Cell Line

The myoblast cell line derived from mouse satellite cells C2C12 (ATCC, USA) was used to obtain functional myotubes. The cells were maintained for no more than 14 passes, at 37 °C with a 5% CO_2_ atmosphere in growth medium composed of DMEM supplemented with 10% fetal bovine serum (FBS), 1M HEPES, 100 units/mL penicillin, and 100 µM streptomycin (DMEM-MM). When cell densities reached approximately 70–80% confluence, C2C12 cells were detached with trypsin and were either replated or used in experiments. A group of C2C12 cells at 70% of confluence were infected with channelrodopsin 2 (ChR2) expressing lentivirus (pLenti-EF1α-hChR2(H134R)-EYFP-WPRE) containing an eYFP (enhanced yellow fluorescent protein) sequence as reporter. The added lentivirus was maintained for 24 h, and infected cells were purified through fluorescence-activated cell sorting (FACS, Scientific services, University of Barcelona) obtaining C2C12-ChR2-positive cells. Isolated cells were seeded and maintained in DMEN-MM. The expression of ChR2 allowed us to specifically stimulate C2C12-ChR2-expressing cells using light, avoiding the use of the electrical stimulation that might affect both C2C12 myotubes and cultured spinal cord slices in our co-cultures.

### 2.4. C2C12 Myotube Differentiation in Microfluidic Platforms

Either C2C12 or C2C12-ChR2 cells were resuspended in pure Matrigel^TM^. Shortly after gentle trypsinization and quantification, 250,000 cells were seeded by gentle injection into one chamber of the microfluidic device and then maintained in a cell culture incubator (37 °C, 5% CO_2_) for 15 min to allow Matrigel^TM^ jellification. Once seeded, C2C12 cells were cultured for 2 days with DMEM-MM. Myoblasts were induced to differentiate into myotubes, facilitated by using differentiation medium (C2C12-DM) consisting of DMEM containing 2% normal horse serum (NHS), 100 units/mL penicillin, 100 µM streptomycin, and 1M HEPES. C2C12-DM was added to all reservoirs, including the axotomy channel, and then renewed every 24 h.

### 2.5. Spinal Cord Explant Cultures in Compartmentalized Microfluidic Devices

Pregnant E12.5 CD1 mice (Charles River Laboratories) were anaesthetized and euthanized by cervical dislocation. Embryos were removed and transferred to a petri dish (10 cm ∅) containing cold (4 °C) glucose-HEPES buffered saline (GHEBS; 137 mM NaCl, 2.7 mM KCl, 22.2 mM glucose, 25 mm HEPES, pH 7.4) solution. Each isolated embryo was rinsed in GHEBS medium and then decapitated. Spinal cords were isolated from the rest of the embryo under dissecting microscope with dark field optics (Olympus, Japan). Blood vessels, muscles, and connective tissue were then removed so that only the spinal cord was left. The spinal cord was cut into 100–125 µm sections using a tissue chopper (Vibratome, UK). Next, slices were hemisected through the middle axis into two lobes and the ventral horns of each lobe were isolated from the dorsal portion, leaving small pieces of ≈60–70 × 100–125 µm (see [12,37]). When required, ventral spinal cord pieces were infected for 18 h with ≈10^11^ GC/mL viral dilution of AAV9.hSyn.hChR2(H134R)-eYFP.WPRE.hGH (uPenn Vector Core, Addgene, USA) in Neurobasal™ medium (ThermoFisher Scientific). The neuronal expression of ChR2 was confirmed by visualization of eYFP fluorescence in an IX71 inverted microscope (Olympus). In parallel, some slides were also infected following similar protocols to those described above with the genetically encoded calcium indicator (GECI) jRCaMP1b (AAV9.Syn.NES-jRCaMP1b.WPRE.SV40, uPenn Vector Core). During experiments, 2-3 ventral horn pieces were cultured per chamber embedded in Matrigel™ and the devices were placed in the incubator for matrix gelation, after which MN medium based on Neurobasal™ medium supplemented with 2% NHS (ThermoFisher Scientific), 2% B27 (ThermoFisher Scientific, Spain), 100 units/mL penicillin, 100 µM streptomycin, 0.5% glutamine (ThermoFisher Scientific), 10 ng/ml BDNF (Preprotech, UK), 10 ng/ml GDNF (Preprotech), and 10 ng/mL ciliary neurotrophic factor (CNTF, Preprotech) was added to the device. One day later, 5 µM of cytosine arabinose (Ara-C; Sigma-Aldrich, UK) was added to the medium to prevent excessive glial cell proliferation. Explants were maintained at 37 °C in a humidified 5% CO_2_ cell culture incubator. Cultures were examined every day, and mediums refreshed daily. 

### 2.6. In Vitro Axotomy in Microfluidic Platforms

Once MN axons had reached the distal (muscle) chamber (≈11–15 days in vitro (DIV)), a vacuum-assisted axotomy was performed. Briefly, the medium inside the axotomy channel was aspirated using a P20 micropipette set at 15 µL, forming a tight seal between the tip and the outlet well (Ø = 1.25 mm). Air bubbles were introduced inside the axotomy channel and fresh medium was then reintroduced through the inlet well. This process was repeated until all axon bundles were removed from the channel, typically after 3–5 repetitions. Once the success of the axotomy was verified on microscope, the medium was removed and pure Matrigel™ was introduced into the axotomy channel. Microfluidic devices (MFDs) were then placed in cell culture incubation for 15 min for Matrigel^TM^ jellification, and then MN medium was added to the inlets. MFDs with equivalent viable cell populations were randomly chosen for either axotomy or unstimulated control groups.

### 2.7. Optogenetic Setup and Stimulation Procedures

Optical stimulation on ChR2-expressing MNs or C2C12-ChR2 positive myotubes was performed using a homemade 6 × 470 nm LED array (SinkPAD-II™ 23mm Quad LED Modules, Luxeon, Canada), under the pulse with modulation (PWM) control of 600 mA Fentobuck drivers (Sparkfun, USA). The Quad LED array was mounted in a 3D-printed culture incubator based on the light plate apparatus (LPA) described in [38] (please see http://taborlab.rice.edu/hardware for the original design for the 24-well plate). Square pulses were generated with an Arduino-UNO^TM^ microcontroller pulse generator with an LCD keypad and a custom-made code based on [39] (see also https://pharmacology.ucdenver.edu/tucker/reagents.html; Supplementary Figure S1) or, alternatively, using a PulserPlus generator and Pulser v3.1 software (Prizmatrix, Israel). During experiments, temperature changes inside the optogenetic platform were monitored by an Arduino-UNO™ microcontroller using a temperature probe DS18B20 with PWM relay output (CEBEK I-86, Spain) connected to a 12 V cooling fan. The cooling fan switched on with temperature increases >0.5 °C from 37 °C. The LED array was placed onto aluminum heat sink plates below the culture dishes in the optogenetic platform at a distance of ≈2 cm to ensure that the complete area of the microfluidic device was illuminated. Illumination was performed through the glass surface (bottom) in order to avoid plastic light diffraction, 30 min and 24 h after axotomy. Alternatively, and only for microscope-associated purposes of optical stimulation (i.e., Ca^2+^ imaging, generation of responses in C2C12 cells after MN-ChR2 stimulation and time-lapse analysis), a 470 nm light source with high power fiber-coupled led was used (Prizmatrix). An optical fiber (NA = 0.50) and a collimator with a focusing module (Focal length 10 mm; Prizmatrix) were used for the optical stimulation. The *xyz* position of the optical fiber was controlled using a 3-axis micromanipulator (Nashirigue, Japan) to specific regions of the platform. ChR2 stimulation pulses (in the culture plates or through the optical fiber) were triggered for 30 min. Square waveform pulse duration was 5 ms, pulse frequency 20 Hz, train duration (stimulation) 1 s, and intertrain duration (non-stimulated) 1 s. In these conditions, the external stimulation voltage unit drives 12 V and 600 mA for each Quad LED module, with an average light intensity of approximately 20–25 mW/cm^2^ measured at the culture dish containing the devices measured with a Newport 1919 optical power meter (Newport Photonics, USA). Alternatively, myotubes were stimulated by an electrical field using an electrical stimulator A-M Systems 2100 model (A-M Systems, USA). Platinum electrodes were fixed in the reservoirs of the microfluidic device on each side of the C2C12 chamber. Stimulation was performed with monophasic square waveform electric pulses of ≈10 V at a frequency of 1 Hz with a pulse duration of 20 ms (8–10 s stimulation/ 8–10 s non-stimulation cycles). Temperature was monitored with a temperature probe immersed in a mock plate without cells during the stimulations; we did not observe an increase of more than 0.3–0.7 °C in any of the experiments. For the experiments, the selected areas of the C2C12-ChR2 myotube chamber was checked 3–4 h before the stimulation and the recordings.

### 2.8. Analysis of ChR2-Positive Activity in Spinal Cord Slides with mRuby-Based RCaMP (jRCaMP1b)

E12.5 spinal cord ventral horns were obtained as above and infected with ChR2-eYFP and jRCaMP1b-expressing viruses at the same concentrations as indicated above for ChR2 (see above). Three days after infection, cultures were optically illuminated with 470 nm light pulses using the optical light fiber system, and changes in the red fluorescence were recorded using a BrightLine^TM^ filter: excitation FF01-554/23-25, emission FF01-609/54-25 filter cube and a Hamamatsu Orca Flash 4.0 camera through an Olympus IX71 inverted microscope with a 20× objective. Time-lapse images (1024 × 1024 pixels) were collected at 20 fps and integrated in Fiji™ in a single TIFF stack for further analysis. Several regions of interest (ROIs, eYFP-, and mRuby-positive) were selected, and the fluorescence trace *F(t)* was normalized nF(t) for each ROI to correct for its background brightness level by computing n*F*(*t*) = (*F(t*)−*F*_0_)/*F*_0_ ≡ Δ*F*/*F*_0_, where *F*_0_ is the average amplitude of the background fluorescence signal at rest.

### 2.9. Analysis of Myotube Contractility

The contractility of the myotubes was also monitored using a Hamamatsu Orca Flash 4.0 camera through an Olympus IX71 inverted microscope with a 20× objective. Time-lapse images (1024 × 1024 pixels) were collected at 40 fps and integrated in Fiji™ in a single TIFF stack for further analysis. To estimate the range of displacement and therefore the contractility of the cells, samples were processed by Matlab™ 2017b running in a Dell workstation. Matlab™ libraries were kindly provided by Dr. Nathaliel Huesbch (Dept. Biomedical Engineering, Washington University in St. Louis) [40]. With the software, among other parameters, the displacement/contraction or the contraction speed of selected ROIs containing C2C12 cells in the same optical plane over time were plotted and the average displacement (AD) in each experiment calculated. In order to do this, for reference purposes, the pixel size, fps, and size of the picture (1024 × 1024) were always the same. However, for the supplementary videos, portions of the analyzed videos were selected to better illustrate the presented data. All experiments were performed in triplicate unless specified. Videos were mounted using Final Cut Pro^TM^ software (iMac OSX 10.14.5).

### 2.10. Immunocytochemical Techniques

Cells were fixed in 4% buffered-paraformaldehyde (PFA) and permeabilized with 0.5% Triton X-100 in 0.1 M phosphate-buffered saline (PBS, pH 7.2–7.3). Before primary antibody incubation, cells were blocked with 10% Normal horse serum (NHS), 0.5% Triton X-100 in 0.1M PBS containing 0.2% gelatin. Primary antibody was diluted in blocking solution and incubated at 4 °C for 72 h to enable the antibodies to enter the Matrigel^TM^. After several washes with 0.1 M PBS–0.5% Triton X-100, secondary antibody was prepared in blocking solution with 5% NHS. Primary antibodies used in this study included anti-neurofilament H (SMI31; 1:500, Chemicon, USA), anti-f-MHC (1:400, Sigma Aldrich), anti-green fluorescence eprotein (GFP; 1:1000 ThermoFisher Scientific), and anti-choline acetyltyransferase (ChAT; (1:1000, Chemicon). Secondary antibodies used at 1:1000 dilution were goat anti-rabbit Alexa Fluor 488 (ThermoFisher Scientific), goat anti-mouse Alexa Fluor-568 (ThermoFisher Scientific), and donkey anti-rabbit Alexa-Fluor-350 (ThermoFisher Scientific). To label the acetylcholinesterase receptor (AChR) clusters, samples were incubated with bungarotoxin (BTX) conjugated with Alexa Fluor-595 (BTX-Alexa Fluor-595, 1:200, ThermoFisher Scientific). Cultures were imaged with an Olympus IX71 inverted microscope and a Hamamatsu Orca Flash 4.0 CMOS cooled camera. Three dimensional and *z*-axis projections were imaged using a Zeiss LSCM-800 confocal microscope and reconstructed using Zen^TM^ v2.5 software (blue edition) and analyzed in Bitplane Imaris^TM^ v7.4 software running on a Dell workstation. The expression of ChAT was used as a marker for MNs located in the ventral horn of the spinal cord in the present study. During the dissection, only the ventral halves of the lumbar spinal cord were cultured, where MNs are present and are predominant. While a proportion of spinal interneurons and autonomic preganglionic neurons can also be ChA+, they are more enriched in the dorsal half and/or thoracic and cervical sections of the spinal cord [41]. Also, these neurons differentiate and emerge at later embryonic stages, especially in the lumbar ventral horns (from E13.5; [42,43]), than those used in our experiments (E12.5).

### 2.11. Quantitative Real-Time PCR

Myotubes were harvested from microfluidic chambers using Corning Cell recovery solution (Corning, USA) for 10 min and left for 1 h on ice until Matrigel was dissolved. Total RNA was then extracted by pooling 2 devices and using an RNeasy kit (Qiagen, USA), according to the manufacturer’s guidelines. cDNA was then synthesized with SuperScript™ II reverse transcriptase (ThermoFisher Scientific) from 1 mg of RNA per sample. Real-time qPCR was run with Light cycler 480 SYBR Green Master (Roche, Spain) in a StepONEPlus light cycler (ThermoFisher Scientific). C_t_s were calculated following the manufacturer’s instructions. Expression values are expressed as 2^-ΔΔCt^. First C_t_s were normalized versus glyceraldehyde-3-phosphate dehydrogenase (*GAPDH*) as a housekeeping gene, and then the relative amount was normalized against corresponding non-stimulated controls. Primer sequences used are described in Table 1.

### 2.12. Functional Roles of Selected Candidates in Spinal Cord Explants

E12.5 ventral spinal cord horns were extracted, as described above. Tissue pieces were seeded onto the bottom of a 35 mm Ø glass plate (Nunc^TM^), on top of a thin layer of Matrigel™ previously mixed with recombinant VEGF (50 ng/mL; Peprotech), LIF (1 ng/mL~10^4^ U/mL; Merck Millipore, Germany), GDNF (30 ng/mL, Peprotech), or Angiopoietin I (100 ng/mL; R&D Systems, USA), or a combination of these. Angpt1 was complexed overnight with a 1:100 dilution of an AntiHis antibody (R&D Systems) before adding it to the Matrigel^TM^. After that, a 20 µL drop of the corresponding Matrigel™ mixture was placed on top of the explant and left at 37 °C for jellification. After 15’, MN culture media without factors (BDNF, CTNF, and GDNF) was added to each plate. Photomicrographs were taken 24 h after seeding with an Olympus IX71 inverted microscope and a Hamamatsu Orca Flash 4.0 camera.

### 2.13. Quantification and Statistical Analysis

Time-course experiments with raw data derived from the Matlab^TM^ scripts or individual axons quantified using the plugin NeuronJ [44] of Fiji^TM^ at different time-points were processed for statistical significance. All data analysis was performed and analyzed blind to experimental groups. Unless otherwise stated, data are plotted as the mean ± S.D. All experiments were performed three times unless specified. Normality of the distributions was checked via the Shapiro–Wilk test; asterisks indicate a significant difference analyzed with ANOVA with Bonferroni post-hoc test or Student’s *t*-test, as indicated (* *p* < 0.05; ** *p* < 0.01; *** *p* < 0.001; **** *p* < 0.0001). All tests performed were two-sided, and adjustments for multiple comparisons and/or significantly different variances (Fisher’s F) were applied as indicated. All data analysis was performed blind to the experimental group. Unless otherwise stated, sample size was chosen in order to ensure a power of at least 0.8, with a type I error threshold of 0.05, in view of the minimum effect size that was expected.

## 3. Results

### 3.1. Design and Fabrication of the NMJ Microfluidic Axotomy/Co-Culture Platform

To develop the main objectives of these studies, we generated a combined compartmentalized microfluidic device suitable for axotomy, optogenetic interventions, and microscopic monitoring to study the role of neuromuscular activity modulation after axotomy of MNs (Figure 1). The device consisted of two main chambers interconnected with 100 microchannels (10 × 10 µm cross-section and 900 µm length). In addition, a transversal axotomy-designed channel was added in the middle of the microchannels (see [34] for details of a similar device). In our experiments, we generated and differentiated ChR2-expressing C2C12 myoblasts into functional myotubes in one chamber before seeding the embryonic spinal cord explants in the other chamber of the device with the appropriate culture media added in the reservoirs (Figure 1a,b). After allowing 11–15 days to ensure that MN axons elongated and crossed the microchannels establishing NMJs with cultured C2C12-ChR2 myotubes, we performed a vacuum-assisted axotomy, and optogenetic stimulation using an LED module array composed of 6 LED arrays containing 4 high power LEDs each (λ = 470 nm) mounted on a 3D-printed illumination platform (see Material and Methods for details, Supplementary Figure S1a). LED activity was controlled with an external TTL source and a homemade circuit including LED drivers and an independent refrigerating relay-controlled fan with a temperature probe (Figure S1b–d). Changes in culture medium temperature during the stimulation were monitored in selected cultures with λ = 470 nm and λ = 590 nm light, and the oscillations of temperature were negligible <0.3–0.7 °C compared to the control non-illuminated devices. Immunocytochemical evaluation of markers of MN (SMI31; Figure 1c) or C2C12-ChR2 myotubes (MHC; Figure 1d) demonstrated the correct on-device differentiation of the spinal cord explants and differentiated myotubes, respectively, after 15 DIV. 

### 3.2. C2C12-ChR2 Myoblasts Differentiate into Light-Inducible Contractile Myotubes

**Figure 2:**
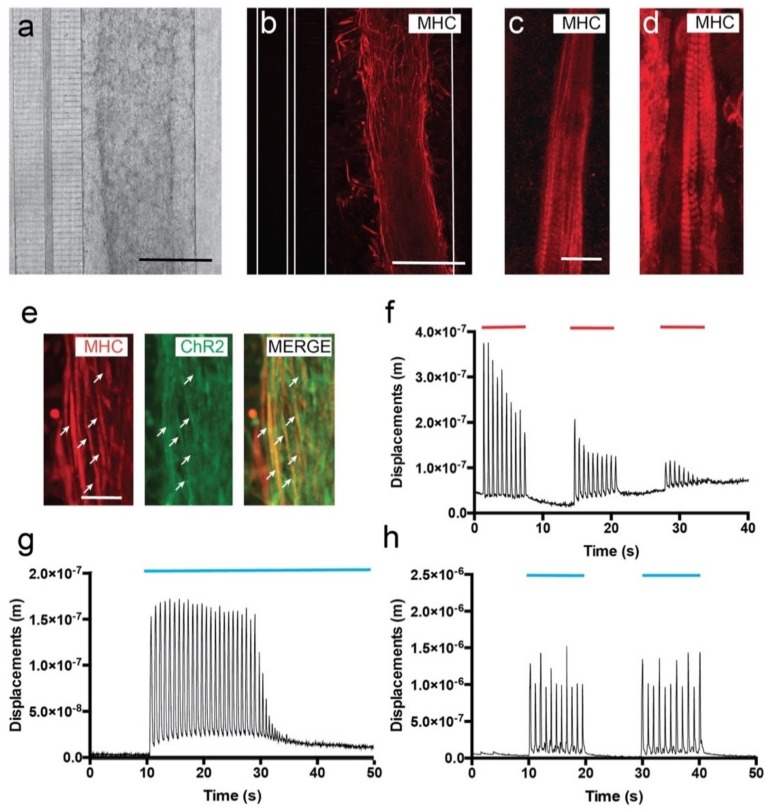
Development and functional properties of cultured C2C12-ChR2 cells in microfluidic devices. (**a**) Low magnification phase contrast (**a**) and fluorescence after the immunohistochemical detection of the MHC (**b**) micrographs illustrating differentiating C2C12 myoblasts in the right chamber 3 days after seeding. As a result of the fixation and the immunohistochemical procedure, a shrinkage of the hydrogel could be observed. Please note that the connecting microchannels and the transversal axotomy channel can be seen on the left in (**a**). The microfluidic limits of these microchannels are labelled as white lines in (**b**). (**c**,**d**) Myotube differentiation of C2C12-ChR2 myoblasts at different in vitro timepoints: 3 (**c**) and 7 days (**d**). Note the increasing presence of the MHC-positive labelling of sarcomers in the differentiating myotubes in (**d**) with respect to (**c**). (**e**) Fluorescence photomicrographs illustrating double-labelled (ChR2-MHC, arrows) differentiating myotubes in the chamber. (**f**) Graph illustrating one example of the displacement measurements (*y*-axis) of differentiated C2C12-ChR2 myotubes after electrical activation (red lines) over time (*x*-axis). (**g**,**h**) Graphs illustrating one example of the displacement measurements (*y*-axis) of differentiated C2C12-ChR2 myotubes after optogenetic activation (blue) in continuous (**g**) or pulsatile (**h**) illumination (blue lines in g and h) (see Material and Methods for details of analysis and Results for the statistical analysis). Scale bars: (a,b) = 100 µm, (c) = 50 µm, (e) = 25 µm.

C2C12 myoblasts were differentiated in the microfluidic device into myotubes; 24 h after seeding, we already observed a progressive orientation of cells (Figure 2a) starting to express MHC (Figure 2b). A patterned sarcomeric MHC staining was already visible in some myotubes (Figure 2c). However, by 7 DIV, >90% myotubes were already differentiated and could easily be identified by their patterned MHC staining (Figure 2d, Supplementary Video S1). Relevantly, ChR2-eYFP expression in differentiated C2C12-ChR2 myotubes was maintained during differentiation (Figure 2e). Concerning myotube orientation, ≈70–75% of the generated C2C12-ChR2 myotubes were spontaneously oriented, mainly perpendicular to the microchannels at 7 DIV. However, in some cases, differentiated myotubes were not perpendicularly oriented in the chamber and other orientations were observed (see Supplementary Video S2 as an example of the various myotube orientations). After myotube formation and microscopic characterization, we started to evaluate the functional contractibility of the generated C2C12-ChR2 myotubes. To this end, we first performed an electrical field stimulation of cultures followed by experiments with optical stimulation (see below). The relative displacement/contraction of identified myotubes was measured (see Material and Methods for details). Analyzed fields of the C2C12-ChR2 cultures were located in central and lateral regions of the differentiated strip of myotubes. In fact, in our experiments, myotube displacements were slightly more robust and 3–4 times higher in the periphery of the cultured cells than in internal portions of the culture due to the increased cell density, as also reported in other studies [45]. In all C2C12 cultures, some myotubes showed spontaneous contraction (scC2C12 cells) (see Supplementary Video S2 as an example). To clearly define changes after electrical/optical induction and to avoid putative baseline changes between experiments, displacements in scC2C12 cell contraction were not evaluated in our experiments, and only the contraction changes induced after electrical/optical stimulation in non-spontaneous contractile C2C12 (nscC2C12 cells) were quantified (see Supplementary Video S2 as an example). For example, in Supplementary Video S2, scC2C12 cells located in ROI1 were ruled out for analysis and only ROIs 2 and 3 (containing nscC2C12 cells) were analyzed. The plot shown in the video corresponds to ROI3. 

The main objective of the electrical stimulation was to determine whether the differentiated myotubes decreased their contraction in the device after several rounds of stimulation, as illustrated in other devices. Electrical stimulation of C2C12 myotubes consisted of long train pulses (20 ms ON and 980 ms OFF of ≈10 V, 1 Hz of frequency). This stimulation protocol was performed for 8–10 s. After this train of stimulation, the electrical stimulation stopped for an additional 8–10 s. In the experiments, 3 cycles of stimulation/non-stimulation were performed until complete exhaustion of the contraction was reached (Figure 2f, Supplementary Video S2). Video time-lapse recordings during stimulation and computer-assisted analysis demonstrated that spikes of contraction matched stimulation pulses, and as expected, the amplitude of displacement (AD) decreased gradually (from AD = 3.636 × 10^−7^ ± 0.4 × 10^−7^ m to 1.633 × 10^−7^ ± 0.5 × 10^−7^ m, mean ± S.D.; ≈75% reduction) in the first train pulses of Figure 2f until complete exhaustion due to myotube fatigue under protracted electric stimulation (last displacement: AD = 7.25 × 10^−8^ ± 0.1 × 10^−8^ m; mean ± S.D., in the third train in Figure 2f; see also Supplementary Video S2). Next, we checked the proneness to optical stimulation of the ChR2-expressing myotubes. First, we stimulated the myotubes with continuous 470 nm light for 40 s (Figure 2g, Supplementary Video S3). Quantification of the AD demonstrated the optical susceptibility of the C2C12-ChR2 myotubes; induced contraction frequency was ≈1.15 Hz, (AD = 1.54 × 10^−7^ ± 0.22 × 10^−7^ m; mean ± S.D.). Stimulated myotubes were exhausted after ≈ 25–30 constant contractions (at ≈70 b.p.m during illumination), similarly to what we observed previously with pulsed electrical stimulation (Figure 2f,g). This fatigue induction during optical and electrical stimulation has also been described in other studies (i.e., [17,46,47]), lending support to our present data. Finally, and in order to avoid myotube exhaustion, we tested their contractibility when subjected to pulsed 470 nm light (see Material and Methods, Figure 2h). After pulsatile light train stimulation, the AD levels of nscC2C12-ChR2 myotubes were increased by an AD of 1.13 × 10^−6^ ± 0.52 × 10^−6^ m, (mean ± S.D.) but with similar frequency to that in continuous optical stimulation (≈1.13 Hz; Figure 2h). Contraction spikes measured through AD matched the cycles of stimulation (Figure 2h, Supplementary Video S4) and allowed the myotubes to recover between spikes, thereby avoiding muscular fatigue and exhaustion (Figure 2h, Supplementary Video S4). 

### 3.3. Viable Co-Culture and Formation of NMJ in Compartmentalized Microfluidic Devices

**Figure 3:**
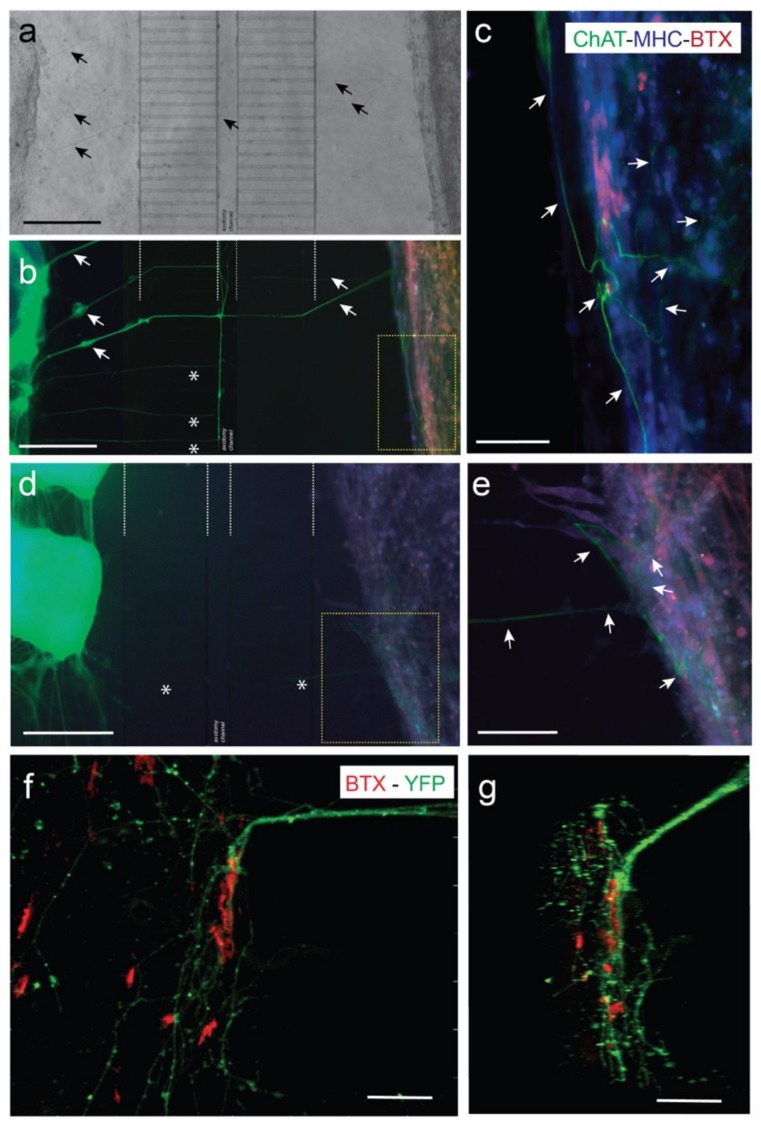
Examples of NMJ formation in our microfluidic devices (MFDs). (**a**,**b**) A low power photomicrograph of the axotomy-assisted device is shown in (**a**). A parallel triple fluorescence image after the immunohistochemical (IH) protocol of the device shown in (**a**) can be seen in (**b**). A high magnification image of the yellow square in (**b**) can be seen in (**c**). This device was immunostained using ChAT antibodies to demonstrate the presence of MN-derived axons in the C2C12 myotube chamber. In addition, cultured cells in the device were also immunostained using MHC antibody (blue) and incubated with BTX-Alexa Fluor-595 (red). (**d**,**e**) Low (**d**) and high (**e**) magnification photomicrographs of a parallel device similar to (**a**–**c**), using the same antibodies. In this example, two pieces of the ventral horns (left) of the dissected spinal cord can easily be observed in (d). Arrows (black in a) and (white in (b)) are included as reference marks between the two images. In addition, asterisks in (**b**,**d**) also label reference microchannels. Arrows (white in (c,e)) point to ChAT-positive axons entering into the C2C12 myotubes. (**f**,**g**) Confocal microscopy images of double labelled (BTX-Alexa Fluor-595 and eYFP) photomicrograph illustrating the location of the eYFP-labelled MN axons (expressing ChR2-eYFP) running over the differentiated myotubes showing BTX-Alexa Fluor-595 labeled spots. A z stack animation of the image shown in (**f**) may be observed in Supplementary Video S5. Scale bars: (a,b,d) = 400 mm, (c,e) = 200 mm, (f) = 70 mm, (g) = 30 mm.

Previous experiments demonstrated that fluidically-isolated microfluidic devices enable the co-culture of spinal cord explants and C2C12-derived myotubes in two separate compartments connected by MN axons in open reservoirs [48]. In the current experiments, co-cultured MN and C2C12-derived myotubes showed their typical morphologies, and with high cell viabilities. Corroborating previous studies by our laboratory (i.e., Tong et al. (2015) [45]), axons reached the axotomy channel a few days after culture, crossing towards the C2C12 myotube chamber in 5–7 days. Indeed, after 7–10 days in co-culture with ventral horns of spinal cord slides, distal MN axons reached the myotubes, establishing neuromuscular junctions (NMJs) labelled with BTX-Alexa Fluor-595 to identify acetylcholine receptor (AChR) clusters on myotubes (Figure 3). In Figure 3, two examples of co-cultures in two different devices (ventral spinal horns (left)) and C2C12-myotubes (right) can be seen. In Figure 3c,e, we showed ChAT-positive axons contacting MHC-labelled (blue) myotubes that also contained clusters of AChR clusters (BTX-positive, red). In the same figure, we also showed examples of eYFP-positive ChR2-eYFP-expressing MN axons contacting BTX-Alexa Fluor-595-positive regions of differentiated C2C12 myotubes (Figure 3f,g, see also Supplementary Video S5). We identified several endings of axons interacting with myotubes forming the well-defined AChR clusters, as also found in previously published data of ours [48] and other laboratories (see Introduction for references). However, these contacts were more often observed in the peripheral portion of the differentiated myotubes than in the internal portion of cultured myotubes, as previously reported [48].

### 3.4. Optogenetic Modulation of MN Activity Increases Axonal Outgrowth after Microfluidic Induced Axotomy 

**Figure 4:**
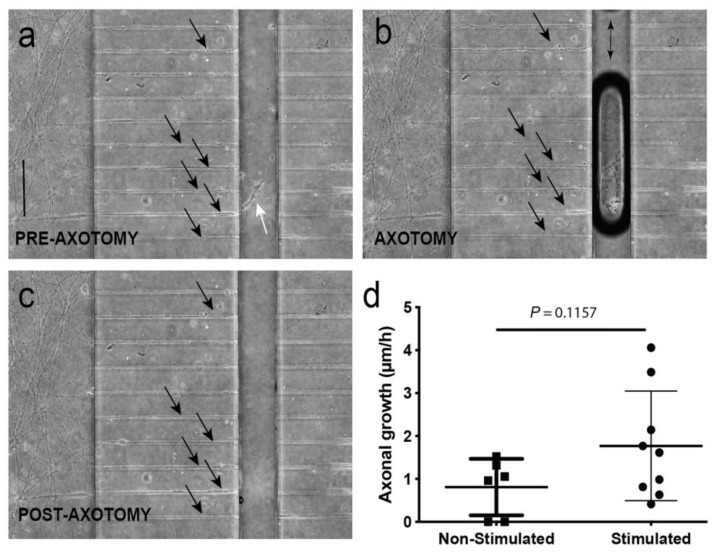
(**a**–**c**) Representative images of the vacuum-assisted axotomy of the motor neuron (MN) axons using the axotomy channel (before (a), during (b), and after (c)) axotomy. Note the presence of the axons inside the channels during the entire procedure (labelled by arrows in (a–c)) showing the absence of a putative fluidic reflux into the channels, as also demonstrated in Tong et al. (2015) [34] using a similar device with identical channel geometry and fluidic resistance. In addition, note the axotomy of the axons labelled with white arrows (a) located in the axotomy channels and their absence in (c). (**d**) Graph illustrating the mean speed mm/h of identified axons in the axotomy channel between axotomy and 48 h in non-stimulated and stimulated experiments. The stimulation was performed in the ventral horn slices expressing ChR2 in the neuronal reservoir (see Material and Methods for details). Data in (d) are represented as mean ± S.D. The *p*-value of the statistical analysis is also displayed. Scale bars: (a) = 300 µm, pertains to (b,c).

First, we determined whether our optical stimulation was able to induce changes in neuronal activity in the cultured ChR2-positive spinal cord using adeno-associated viral infection of jRCaMP1b in ChR2-expressing spinal cord slides (Supplementary Figure S2). Results showed clear synchronicity in transient calcium waves measured as fluorescence trace (*ΔF/F0*) changes after 470 nm light pulses in analyzed ROIs of the spinal cord slices (Figure S2b). Next, we determined whether these optically induced changes of activity in the ChCR2-positive spinal cord slides also triggered parallel myotube contraction in co-cultures (Supplementary Figure S3 and Video S6). In the experiments, we optically stimulated the spinal cord, and the C2C12 myotube (ChR2-negative) contraction measurement was analyzed. We measured groups of myotubes that were in the outermost part of the C2C12 culture where the effects of the stimulation were most evident, corroborating our previous observation of the presence of a large number of BTX-positive NMJs in these regions. As observed in Supplementary Figure S3, 470 nm light pulses induced robust waves of contraction in these C2C12-ChR2 negative myotubes (AD = 3.4 × 10^−6^ ± 0.67 x 10^−6^ m, mean ± S.D.) (see also Supplementary Video S6). This contraction can be blocked by the addition of Tetrodoxin (TTX, 5mM) in the spinal cord chamber (not shown) during optical illumination similarly, as in previous studies of our laboratory [48]. Then, in order to evaluate the effect of neuronal activity after axotomy of MN axons, we first monitored the axotomy process by seeded ChR2-positive spinal cord explants (see Material and Methods for details) and allowed them to grow their axons for 7 days through the microchannels, and proceeded then to perform the axotomy. The axotomy process (in brightfield optics) is summarized in Figure 4 and can be seen in Supplementary Video S7 (in ChR2-eYGP fluorescence axons growing for 15 DIV). In Supplementary Video S7, two examples of the axotomy of ChR2-eYGP fluorescence axons crossing several microchannels are presented. In addition, Figure 4a–c show representative brightfield microscopic images of the perpendicular axotomy channel before (A), during (B), and after (C) the vacuum-assisted axotomy via aspiration using a common laboratory micropipette [34]. After the axotomy, we proceeded to optically stimulate the ChR2-positive spinal cord explants as above (Figure 1a, see also Methods for details). Although the behavior of single identified axons after axotomy was impossible to determine due to the large number of MN axons crossing the microgrooves, we were able to detect an increased regrowth of previously lesioned axons only after the MN optical stimulation by using Calcein^TM^ (Supplementary Figure S4, see also Tong et al. (2015) [34] for technical details). Indeed, after optical stimulation (see Material and Methods) we were able, using phase contrast microscopy, to quantify the regrowth of these axons in parallel devices. We quantified the number of axons that reached the middle portion of the axotomy channel 48 h after the axotomy procedure in stimulated vs. non-stimulated cultures, determining a positive effect (≈2-fold), although not statistically significant, of enhanced axon regrowth (1.770 ± 0.42 µm/h; mean ± S.D.) compared to non-stimulated time-matched controls (0.809 ± 0.26 mm/h; mean ± S.D.; *p* = 0.1157, unpaired *t*-test; Figure 4d). These results are in line with recently published data by Hyung et al. (2019) on MN responses to optical stimulation in 3D hydrogels [49]. 

### 3.5. Optogenetic Modulation of Muscular Activity Induces Paracrine Signaling, Triggering Axonal Growth after Lesion on MNs

In this set of experiments, we first checked whether the axotomy process of the MN axons modified the contraction behavior of nscC2C12-ChR2 negative myotubes (Supplementary Video S8). During the experiment, we induced their contraction by illuminating the ChR2-positive spinal cord explants with 470 nm light, as above. However, we observed that after axotomy, the contraction of identified scC2C12-ChR2 negative myotubes was stopped while the 470 nm light illumination was maintained. (Supplementary Video S8). Next, we wanted to test whether modulating muscular activity might play a role in enhancing axonal regrowth of MN after axotomy. 

**Figure 5:**
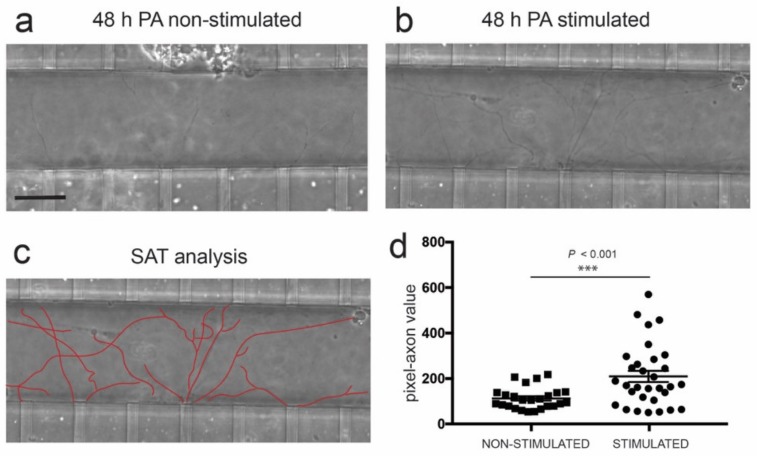
(**a**,**b**) Phase contrast images illustrating spinal cord-derived axons located in the axotomy channel 48 h after axotomy (post-axotomy (PA)) in the absence (**a**) or presence (**b**) of optical stimulation (see Material and Methods for details of the pulsatile optical stimulation) in the C2C12-ChR2 differentiated myotubes. See also Supplementary Figure S4. (**c**) Example of the single axon tracing (SAT) analysis performed in the picture shown in (**b**). This analysis quantifies the number of pixels occupied by the delineated axons. (**d**) Graph illustrating the quantification of the experiments illustrated in (a–c). In the graph, each dot corresponds to the number of pixels occupied by a measured axon. The number of the devices analyzed in these experiments was 5 (stimulated) and 5 (non-stimulated). Plotted data correspond to mean ± S.E.M. The *p*-value of the statistical analysis is included. Scale bar: (a) = 50 µm, pertains to (b,c).

Taking this into account, we co-cultured C2C12-ChR2-positive myotubes with ChR2-negative ventral spinal cord explants in compartmentalized devices, as described (Figure 1). The MN axons were axotomized, and as to the modulation of myotube activity, ChR2-positive myotubes were stimulated 30 min and 24 h after axotomy. After time-lapse recording and computer analysis of spinal cord axon regrowth at 48 h after axotomy, we noted a significant increase in axon regrowth with several trajectories into the axotomy channel after stimulation of C2C12-ChR2 myotubes vs. non-stimulated devices (233 ± 25.54, mean ± S.E.M., pixel-axon value vs. 106.6 ± 9,537, mean ± S.E.M.; *p* < 0.001, ANOVA Bonferroni post hoc test (Figure 5d).

### 3.6. Activity-Dependent Muscle-Derived LIF and GDNF Induce Axonal Growth on MN

**Figure 6:**
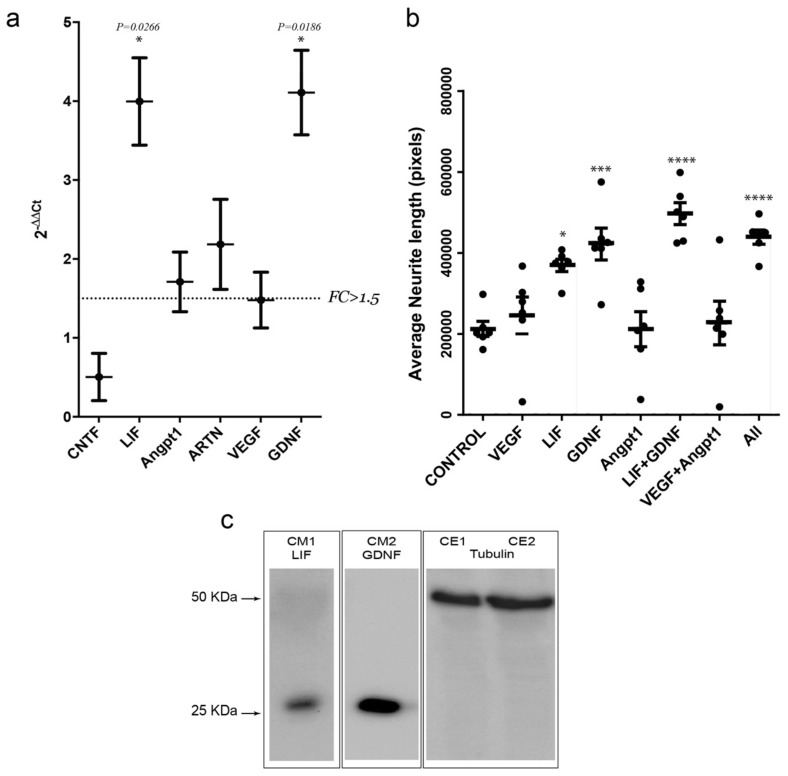
(**a**) Graph illustrating the RT-qPCR data of the selected mRNAs after stimulation of C2C12-ChR2 positive myotubes in the MFDs. The dashed line in (a) marks the threshold of the fold change (FC) used in the statistics (FC > 1.5). (**b**) Graph illustrating the average neurite length of spinal cord explants (*y*-axis) in each treatment condition (*x*-axis). Each dot represents an independent biological replicate. Plotted data correspond to mean ± S.D. Asterisk indicates statistical significance vs. control (* *p* < 0.05; *** *p* < 0.001 and **** *p* < 0.0001). (**c**) Western blot illustrating the presence of LIF and GDNF in the culture media (CM) of two different devices after optical stimulation of the C2C12-ChR2 myotubes. The presence of tubulin in the cell extracts (CE) of the two C2C12-ChR2 positive stimulated cells is also displayed.

Next, we aimed to delve deeper into the putative mechanism by which optogenetic stimulation of muscular activity increases the regenerative capacity of MN after in vitro axotomy. To this end, we first assessed, on stimulated and non-stimulated C2C12-ChR2-positive myotubes, the relative mRNA expression, through RT-qPCR, of several soluble candidate factors that might be involved in the signaling underlying the increase in axonal regrowth. This group includes the interleukin 6 family members *CNTF* and *LIF*, the angiogenic factors vascular endothelial growth factor (*VEGF*) and angiopoietin I (*Angpt1*), the basic fibroblast growth factor (*Fgfb*), *GDNF*, neurturin (*NRTN*) and artemin (*ARTN*), *BDNF*, and neurotrophin-3 (*NT-3*) (Figure 6). Among all these factors, *Fgfb*, *NRTN*, *BDNF,* and *NT3* mRNA expression levels were below the detection threshold (C_T_ > 30). Of the others, only *LIF* and *GDNF* mRNAs showed a robust increase (*p* < 0.05; >1.5-fold increase) in light-stimulated C2C12-ChR2 myotubes when compared to the non-stimulated controls (Figure 6a). In parallel, *Angpt1* and *ARTN* also showed an FC value higher than 1.5 but without statistical significance. 

Lastly, to assess whether these factors could affect the growth rate of MNs axons, we cultured spinal cord slices in Matrigel™ in the presence of recombinant proteins of VEGF, LIF, GDNF, or Angpt1 (Figure 6b) following these approaches: (i) single treatments with either recombinant VEGF, LIF, GDNF, or Angpt1; (ii) a combination of VEGF + Angpt1 or LIF + GDNF; and (iii) all factors together. After quantification of neurite length after 24 h, only LIF and GDNF showed a significant increase in neurite length (measured pixels) when compared to vehicle (369,077 ± 37,191 vs. 212,144 ± 45,641; mean ± S.D, *p* = 0.0233 for LIF; and 421,848 ± 96,243 vs. 212,144 ± 4,5641 mean ± S.D., *p* = 0.0011 for GDNF). In fact, the combination of the two factors (LIF + GDNF) showed an even higher average neurite length (497,040 ± 66,495 vs. 212,144 ± 45,641; mean ± S.D., *p* < 0.0001), comparable to the combination of all factors (438,993 ± 42,573 mean ± S.D. vs. 212,144 ± 45,641 mean ± S.D.; *p* = 0.0004; ANOVA Bonferroni *post hoc* test; Figure 6b).

## 4. Discussion

By taking advantage of an approach combining microfluidics, in vitro axotomy, and optogenetics, this study suggests the previously undescribed role of muscular activity through a paracrine mechanism to promote axonal regrowth of MNs after injury. Specifically, we found that optogenetic modulation of neuronal activity induces a ≈2-fold increase (although non-statistically significant) in axonal regrowth, similar to what was observed by [49]. In addition, we determined that modulating C2C12 contractility after MN axotomy by optogenetics induces an increase in the expression and release of LIF and GDNF, which in turn increases the regrowth of MN axons. This is in line with recent studies reporting that the activity-dependent modulation of C2C12 myotubes is able to enhance angiogenesis in lab-on-chip devices [45,50]. 

While there have been many attempts to reconstruct the NMJ in lab-on-chip devices (see [51,52] for recent reviews), our study involves an approach that combines the use of an axotomy-purposed microfluidic device, which in turn allows compartmentalization of the neuronal and muscular components while allowing axonal manipulation, with optogenetic control of both muscular and neuronal activity. Cultured C2C12-ChR2 cells in our devices embedded in hydrogels displayed contractile properties similar to those seen in similar cultures of C2C12 cells [46,47] (i.e., fatigue after electrical/optical stimulation or contraction properties). Thus, this platform allows us to identify previously undefined pathways and mechanisms underlying the role of neuromuscular activity after axotomy. While neuronal activity, through neurorehabilitative therapies, has long been thought to have beneficial effects on neuronal survival and plasticity after axonal injuries, here it has been systematically demonstrated that specific modulation of the activity of an MN after injury by light will improve their axonal regrowth potential. 

Similarly, the establishment of C2C12-ChR2 myotubes allowed identification of the benefit of promoting contractility onto denervated muscle to promote axonal regrowth on MN through paracrine signaling after injury. While several studies have highlighted the presence and importance of secreted factors from denervated muscle after MN axonal injuries for the regrowth and guidance of these axons [31,32], thus far no data are available on the role of modulating the contractility/activity of this denervated muscle on axon regenerative events in vitro. Thanks to the modularity and specificity achieved by the use of optogenetics, we were able to describe the increase in the axonal regrowth of MNs after lesion, due to the optogenetic modulation of muscular contractility/activity from denervated C2C12-ChR2 myotubes. 

We have also described the paracrine nature of this activity-induced signaling mechanism. Similarly, paracrine activity of cultured myotubes in vitro enhancing angiogenesis has recently been described by R.D. Kamm’s laboratory [45]. In fact, the main muscle-derived factor modulating angiogenesis in the study was Angpt1. Here, we analyzed the expression of different soluble factors that have been described as being expressed in muscle and muscle-like cell lines, as well as being involved in regenerative events in different tissues or neuronal types such as the interleukin 6 family members CNTF and LIF in inducing axonal regeneration on retinal ganglion cells and dorsal root ganglia neurons [53,54,55,56] and the angiogenic factors VEGF and angiopoietin I [57]. The latter has recently been described as being secreted by muscular cells to induce vascularization after injury [45] and was also described as boosting regeneration in dorsal root ganglia (DRGs) [58], Fgfb [57] implicated in the development and regeneration of spinal cord, liver, heart, and photoreceptors from the zebrafish [59]. Moreover, released by the muscle in an autocrine fashion after injury [60] is the glial-derived neurotrophic factor GDNF, whose direct muscle delivery with human mesenchymal stem cells improves motor neuron survival and function in an amyotrophic lateral sclerosis (ALS) model [61]. In addition, motor neuron survival can be promoted by the muscle-specific overexpression of GDNF or exogenous GDNF [62,63,64,65,66,67], which is why in embryonic motor neuron culture GDNF is added as a survival factor. Similarly, exogenous NRTN can also support motor neuron survival [68,69], and ARTN exerts neuroprotective effects on sensory neurons as well as influencing behavioral thermal sensitivity after peripheral transection. However, the effect on motor neuron axon regeneration may result from an indirect effect through Schwann cells in the injured nerve [70,71], as well as the neurotrophins brain-derived neurotrophic factor (BDNF) and neurotrophin-3 (NT-3). Among all these factors, unfortunately, we were not able to detect significant expression of *Fgfb*, *NRTN*, *BDNF,* or *NT3* on our stimulated myotubes. This is in line with the literature demonstrating that expression of BDNF decreases in myotubes and is hardly detectable in mature skeletal myofibers [72]. Our results confirmed the overexpression of Angpt1 after stimulation [45]. However, only LIF and GDNF showed a strong increase after optogenetic stimulation, displaying a ≈4-fold increase in mRNA expression on C2C12-ChR2-derived myotubes. Different studies already highlighted the increase of LIF and GDNF in denervated or injured muscle [73,74,75,76], but no data were presented on the effect of muscular contractility/activity on the expression of these factors. In fact, in a study done with a mouse model of muscular dystrophy (MDX), the authors showed that exercise does increase the expression of LIF, but this does not happen in wild-type mice [77], highlighting the importance of this mechanism after dystrophic or injury events. In the case of GDNF, it has been shown that exercise drives the increase of GDNF expression in skeletal muscles and that this increase is responsible for activity-dependent remodeling of the NMJ [30]. This is in line with our observations demonstrating that the optogenetic stimulation of muscular activity triggers GDNF expression which in turn enhances MN axonal regrowth. 

Consistent with the results found with the expression of the different factors, only LIF and GDNF, and their combination, were able to stimulate the axonal growth of spinal MN when added to the extracellular matrix in vitro. The fact that the combination of the two factors increases the growth capacity even more than each factor alone highlights the different mechanisms by which they act on the MN. There are some studies describing the signaling triggered by GDNF and LIF in axonal regeneration events [32,53,78,79], such as those triggered by LIF/gp130/JAK/STAT in DRG and retinal ganglion cell (RGC) regeneration [54,80], and the non-classical pathway of GDNF RET-independent signaling, which utilizes neural cell adhesion molecule (p140^NCAM^) and its specific receptor GFRα1 to trigger Fyn and focal adhesion kinase (FAK) [81], leading to cytoskeleton remodeling, neurite growth, and cell migration in developing CNS neurons. However, the exact mechanisms by which these two molecules boost axonal growth in spinal MN remain undefined and warrant further studies.

Taken together, our findings show a paracrine mechanism for the regulation of the regenerative ability of MN after axonal injury. Our data reinforce and expand upon the notion of paracrine action of muscle-derived factors in several processes [16,82]. This is relevant since tip cells of growing blood vessels, growing axons, and migrating neurons share several molecular mechanisms modulating their proregenerative activities (i.e., [83,84,85] for reviews). We believe that our approach would be useful not only after axotomy, but also in diseases affecting MN survival.

## Figures and Tables

**Table 1:** Primers used in the present study.

Gene	Forward (5’-3’)	Reverse (5’-3’)
**CNTF**	*TTAGGGGATGGCTTTCGCAG*	*GGAGGTTCTCTTGGAGTCGC*
**LIF**	*GGTGGAGCTGTATCGGATGG*	*ATTGAGCTTGACCTGGAGGC*
**Angpt1**	*GGAACCGAGCCTACTCACAG*	*CAAGCTGCTCTGTTTGCCTG*
**ARTN**	*GAGCCTACTGCATTGTCCCA*	*CAAATGCGCAGTGTGTCCC*
**NRTN**	*CTACACGTCGGATGAGACCG*	*GACACCTCGTCCTCATAGGC*
**BDNF**	*AGTCTCCAGGACAGCAAAGC*	*TCGTCAGACCTCTCGAACCT*
**VEGF**	*GCAGACTATTCAGCGGACTCA*	*GGGAGTGAAGAACCAACCTCC*
**GDNF**	*GCATTCCTGCTACAGTGCGA*	*CACCCTGAAGTGCTCAGACG*
**Fgfb**	*TCAGTCCAGGCACCCTGT*	*GGGGCTCTCTTCACTCCACT*
**NT3**	*GGAAGTCCTTCAAAGGGATCGT*	*GCAGAAGTAACCATGGCATCC*
**GAPDH**	*ACCCTGTTGCTGTAGCCGTATCA*	*TCAACAGCAACTCCCACTCTCCA*

## Supplementary Materials

The following are available online at https://www.mdpi.com/2073-4409/9/2/302/s1, Figure S1: Optogenetic platform illumination; Figure S2: Calcium waves in spinal cord slides after optogenetic stimulation. Figure S3: Quantification of the displacement (µm) of ChR2-C2C12 myotubes under pulsated light stimulation. Figure S4: Axon regrowth in axotomized devices analyzed by Calcein^TM^ labeling. Video S1: Z stack animation of aligned myotubes inside the chamber in the MFD. Video S2: Example of the electrical stimulation on C2C12-ChR2 cultured myotubes. Video S3: Example of continuous optical stimulation on C2C12-ChR2 myotube contractibility. Video S4: Example of pulsatile optical stimulation on C2C12-ChR2 myotube contractibility. Video S5: Z stack animation of double labelled eYFP axons and BTX-Alexa Fluor 595. Video S6: Contraction of C2C12-ChR2-negative myotubes induced by optical stimulation of spinal cord explants. Video S7: Example of vacuum-assisted axotomy in eYFP fluorescence axons. Video S8: Impairment of myotube contraction after axotomy under continuous illumination of ChR2-positive spinal cord explants.
