# Supplementary material for: Neuromuscular Activity Induces Paracrine Signaling and Triggers Axonal Regrowth after Injury in Microfluidic Lab-On-Chip Devices
Source: Cells. 2020 Jan 27;9(2):302. doi: 10.3390/cells9020302 (PMC7072511; doi:10.3390/cells9020302)
Supplement: Supplementary file 1 [file cells-09-00302-s001.zip › Suplementary information Sala-Jarque et al.pdf]

Neuromuscular activity induces paracrine signaling and triggers axonal regrowth after injury in microfluidic lab-on-chip devices

Julia Sala-Jarque[1], Francina Mesquida-Veny[1], Maider Badiola-Mateos[2], Josep Samitier[2], Arnau Hervera[1,*], José Antonio del Río[1,*]

1 Institute for Bioengineering of Catalonia (IBEC), The Barcelona Institute of Science and Technology, Spain. Centro de Investigación Biomédica en Red sobre Enfermedades Neurodegenerativas (CIBERNED), Spain. Department of Cell Biology, Physiology and Immunology, Faculty of Biology, Universitat de Barcelona, Spain. Institute of Neuroscience, University of Barcelona, Spain.

2 Institute for Bioengineering of Catalonia (IBEC), The Barcelona Institute of Science and Technology, Spain. Centro de Investigación Biomédica en Red en Bioingeniería, Biomateriales y Nanomedicina (CIBERBBN), Spain. Department of Electronics and Biomedical Engineering, Universitat de Barcelona, Spain.

* Shared correspondence: ahervera@ibecbarcelona.eu (AH) and jadelrio@ibecbarcelona.eu (JADR).

Supplementary Information

Figure S1. Optogenetic platform illumination. **a)** Photomicrograph showing the illumination platform (left) containing the 6 LED modules and the cooling system (right). **b)** Photomicrograph of the control unit of the platform with the 3 independent LED outputs, the Pulse width modulation (PWM) control input, the temperature probe and the fan output. **c)** Scheme of the assembled system showing the LED layer (blue) the microfluidic devices (orange) and the fan cooler and the air circulation (red arrows). **d)** Summary of the electrical scheme of the different elements of the Arduino UNO R3™ based illumination platform.

Figure S2. Calcium waves in spinal cord slides after optogenetic stimulation. Photomicrograph shows the jRCamP1b fluorescence on a spinal cord ~~cord~~ (SC) explant with the ROIs quantified in the below graph. Normalized jRCamP1b fluorescence changes ($\Delta F/F_0$) are quantified over the duration of the experiment. Calcium transients are observed only during the pulsated stimulation period.

Figure S3. Quantification of the displacement (μm) of C2C12-ChR2 ~~C2C12~~ myotubes under pulsated light stimulation (see Material and methods for details). Below photomicrographs show

in red the force vectors corresponding to the calculated displacement of the myotubes for each frame using the analysis software.

Figure S4. Axon regrowth in axotomized devices analyzed by Calcein™ labeling. **a-d)** Fluorescence photomicrograph of a Calcein™ labelled SC spinal cord axons during the axotomy procedure. Some of these axons crossed the axotomy channel can be seen in (a, red arrows) reaching the distal microchannels microgrooves (red asteriscks in a). The blue arrow points to an unfocussed burble of the device as references in (a-d). Notice that after stimulation some axonal bundles (** in c and d) do not display any outgrowth and their distance (yellow bar) until the axotomy channel remain unchanged. **e-g)** High magnification of the boxed area in (b) at post-axotomy, after 24 h (f) and 48 (g) hours of stimulation. Notice the outgrowth of SC spinal cord axons during the illumination. White arrows in (-b-g) point to reference marks in the deviceMFD.

Video S1. This movie shows a z stack animation of maturated myotubes in the muscle chamber reservoir of the- MFDdevice.

Video S2. Example of the electrical stimulation on cultured C2C12-ChR2 myotubes. Example of a ROI analysis of contractile myotubes after electrical stimulation. The appearance/disappearance of a red sphere illustrates the ON/OFF electrical stimulation (see Material and methods for details). The motion vector illustrating the displacements are showed in red.

Video S3. Example of the analysis of contractile C2C12-ChR2 myotubes with perpendicular orientation after continuous optical illumination. The appearance/disappearance of a blue sphere illustrates the ON/OFF stimulation.

Video S4. Example of the analysis of contractile C2C12-ChR2 myotubes with perpendicular orientation after pulsatile optical illumination. The appearance/disappearance of a blue sphere illustrates the ON/OFF pulsatile optical stimulation.

Video S5. This movie shows a z stack animation of double labelled eYFP axons (green) and BTX-Alexa Fluor-595 (red) differentiated myotubes.

Video S6. Contraction of –C2C12-ChR2 negative myotubes induced by the stimulation of innervating ChR2-positive SC spinal cord explants containing the ventral horns.

Video S7. Examples of vacuum mediated axotomy of eYFP-ChR2 labelled MN axons from SC spinal cord explants on MFDs.

Video S8. Impairment of C2C12-ChR2-negative myotubes contraction under continuous illumination of ChR2-positive SC spinal cord explants.
