# Supplementary figures and images for: Neuromuscular Activity Induces Paracrine Signaling and Triggers Axonal Regrowth after Injury in Microfluidic Lab-On-Chip Devices
Source: Cells. 2020 Jan 27;9(2):302. doi: 10.3390/cells9020302 (PMC7072511; doi:10.3390/cells9020302)

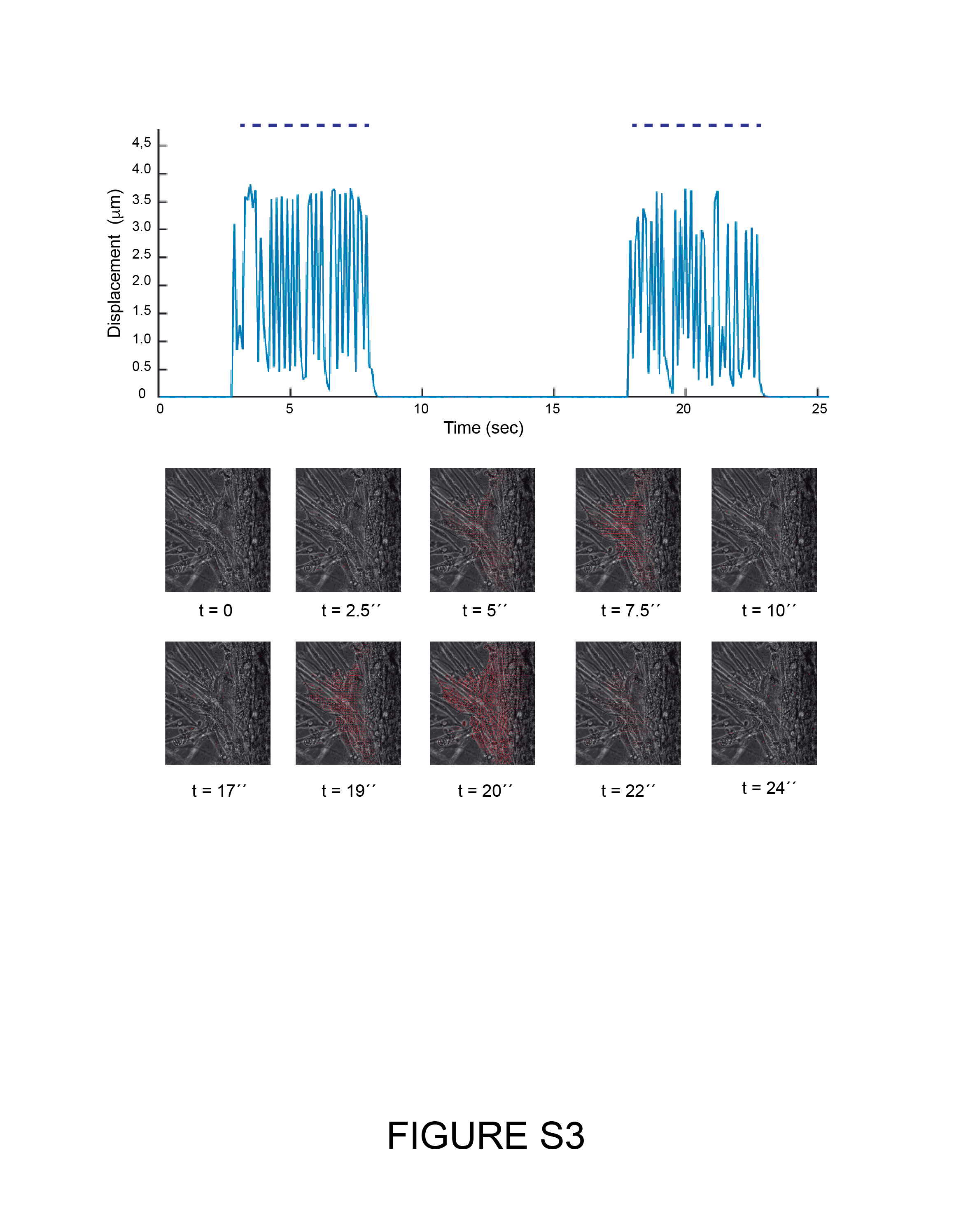

Supplement: Supplementary file 1 [file cells-09-00302-s001.zip › FIGURE S3 Sala_Jarque et al.jpg]

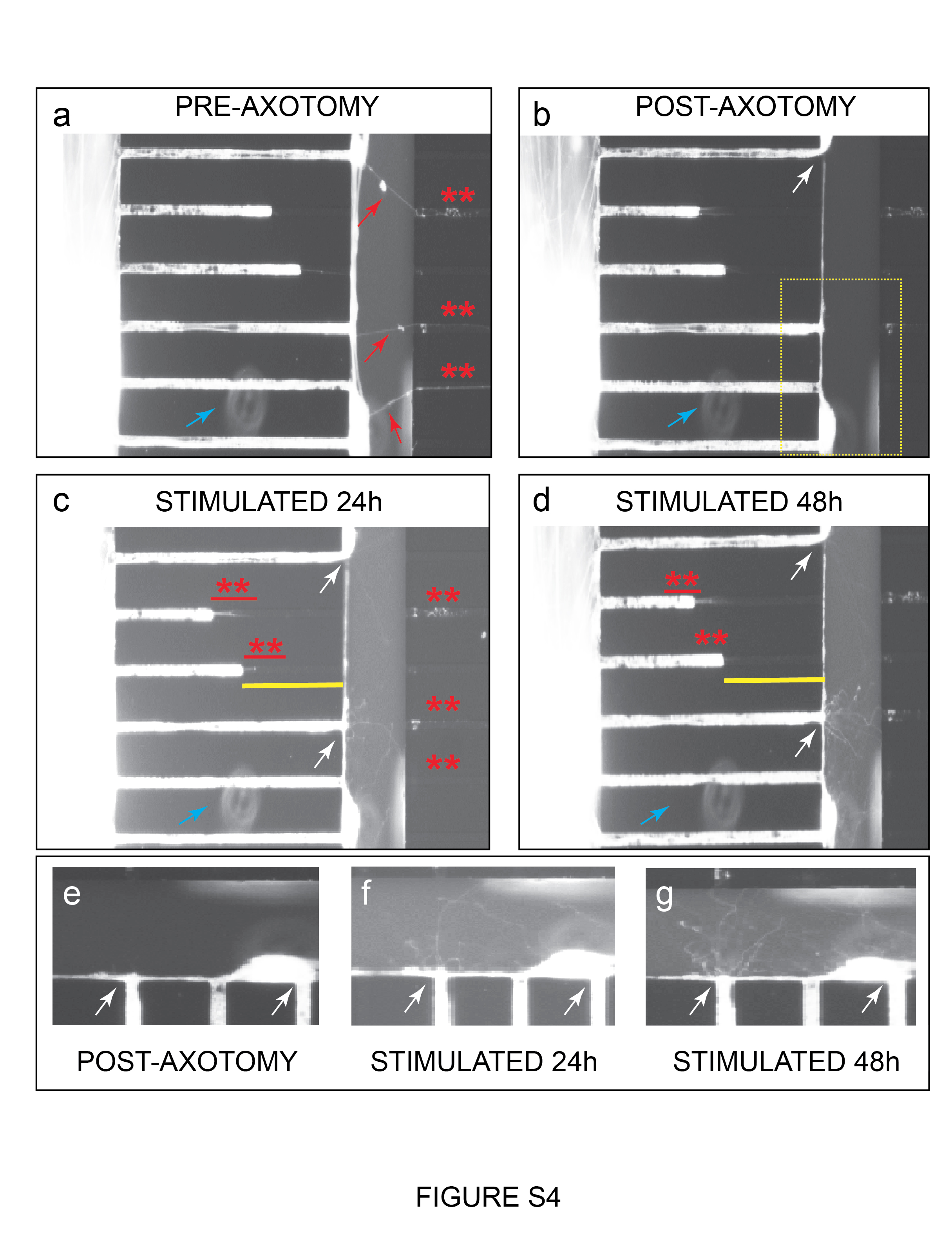

Supplement: Supplementary file 1 [file cells-09-00302-s001.zip › FIGURE S4.jpg]

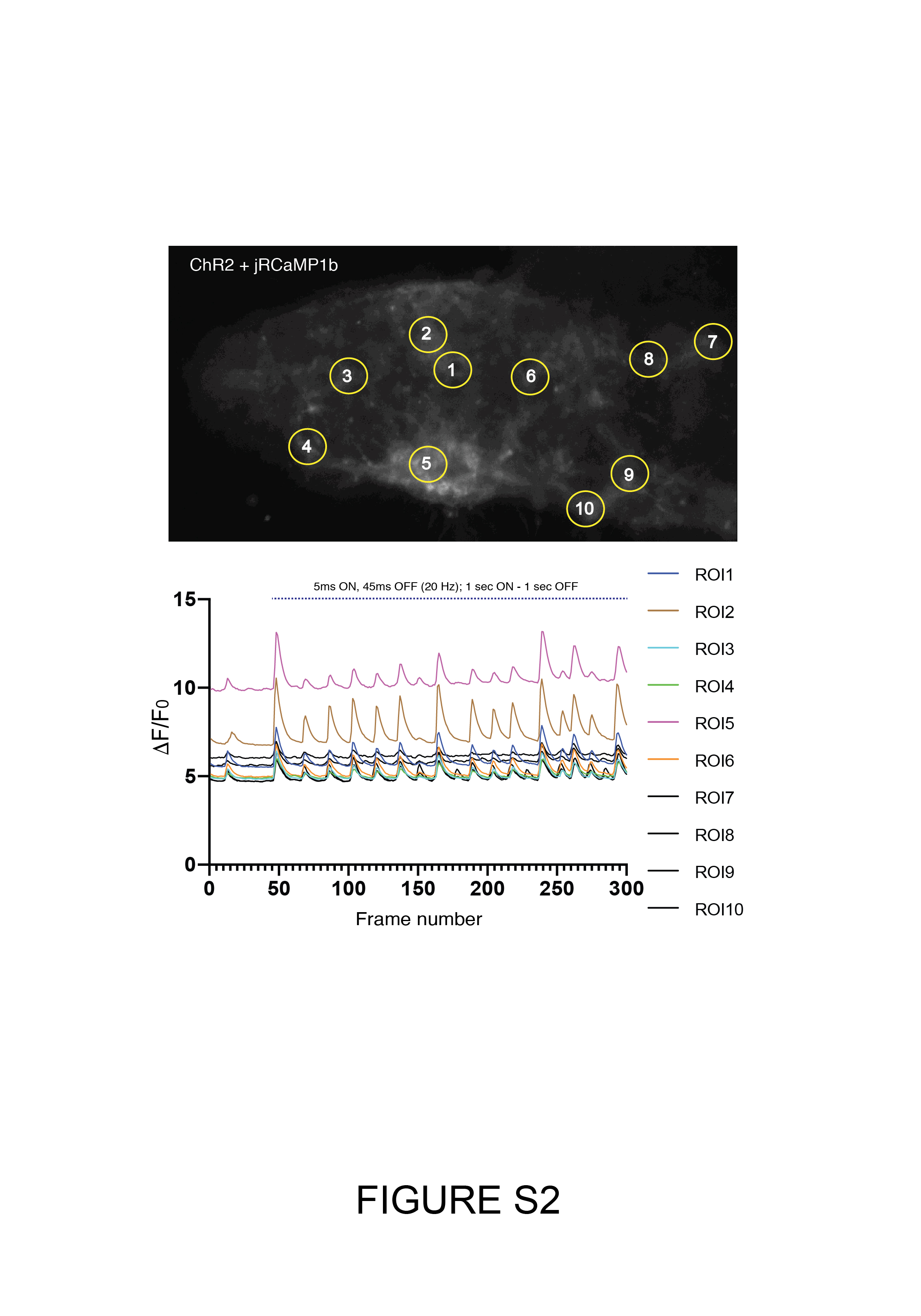

Supplement: Supplementary file 1 [file cells-09-00302-s001.zip › Figure S2 Sala_Jarque et al.jpg]

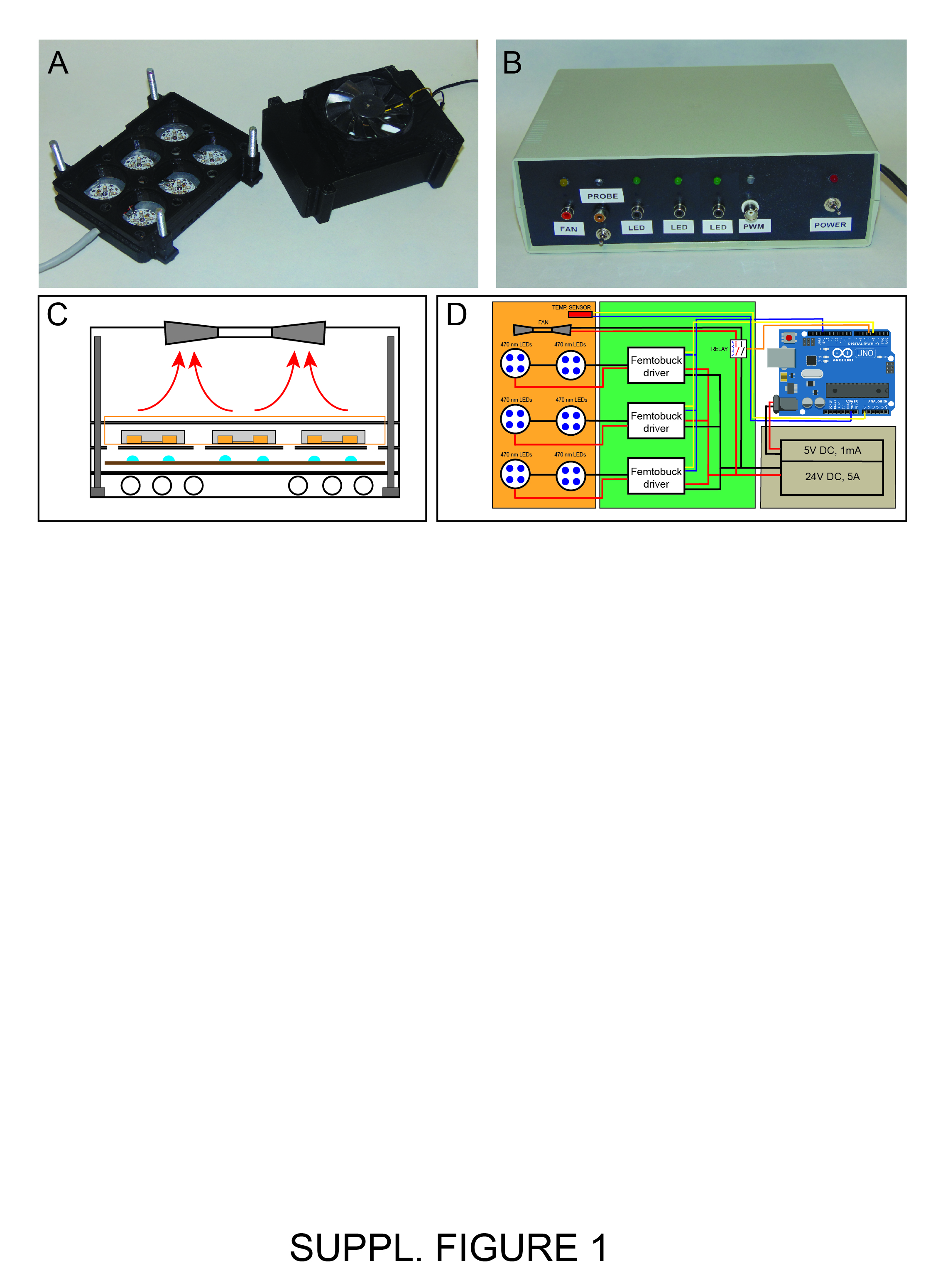

Supplement: Supplementary file 1 [file cells-09-00302-s001.zip › SUPPL Figure 1ai.jpg]
